# Hierarchical organization of objects in scenes is reflected in mental representations of objects

**DOI:** 10.1038/s41598-022-24505-x

**Published:** 2022-11-23

**Authors:** Jacopo Turini, Melissa Le-Hoa Võ

**Affiliations:** 1 Scene Grammar Lab, Department of Psychology and Sports Sciences, Goethe University, Frankfurt am Main, Germany; 2 Scene Grammar Lab, Institut Für Psychologie, PEG, Room 5.G105, Theodor-W.-Adorno Platz 6, 60323 Frankfurt am Main, Germany

**Keywords:** Human behaviour, Object vision

## Abstract

The arrangement of objects in scenes follows certain rules (“Scene Grammar”), which we exploit to perceive and interact efficiently with our environment. We have proposed that Scene Grammar is hierarchically organized: scenes are divided into clusters of objects (“phrases”, e.g., the sink phrase); within every phrase, one object (“anchor”, e.g., the sink) holds strong predictions about identity and position of other objects (“local objects”, e.g., a toothbrush). To investigate if this hierarchy is reflected in the mental representations of objects, we collected pairwise similarity judgments for everyday object pictures and for the corresponding words. Similarity judgments were stronger not only for object pairs appearing in the same scene, but also object pairs appearing within the same phrase of the same scene as opposed to appearing in different phrases of the same scene. Besides, object pairs with the same status in the scenes (i.e., being both anchors or both local objects) were judged as more similar than pairs of different status. Comparing effects between pictures and words, we found similar, significant impact of scene hierarchy on the organization of mental representation of objects, independent of stimulus modality. We conclude that the hierarchical structure of visual environment is incorporated into abstract, domain general mental representations of the world.

## Introduction

Objects in our environment are not arranged randomly but usually appear in certain contexts (“semantic rules”) and in certain positions (“syntactic rules”), according to physical laws and typical use^1^. We refer to this set of rules of objects in scenes as “Scene Grammar” (for a recent review see^2^), in analogy with the linguistic grammar that governs words in sentences. It has been shown that Scene Grammar is exploited by our cognitive system to efficiently represent objects during visual perception and to guide allocation of attention during scene perception^3,4^ supporting complex behaviors like object recognition^5^, search^6^, and object interaction^7^.

**Figure 1:**
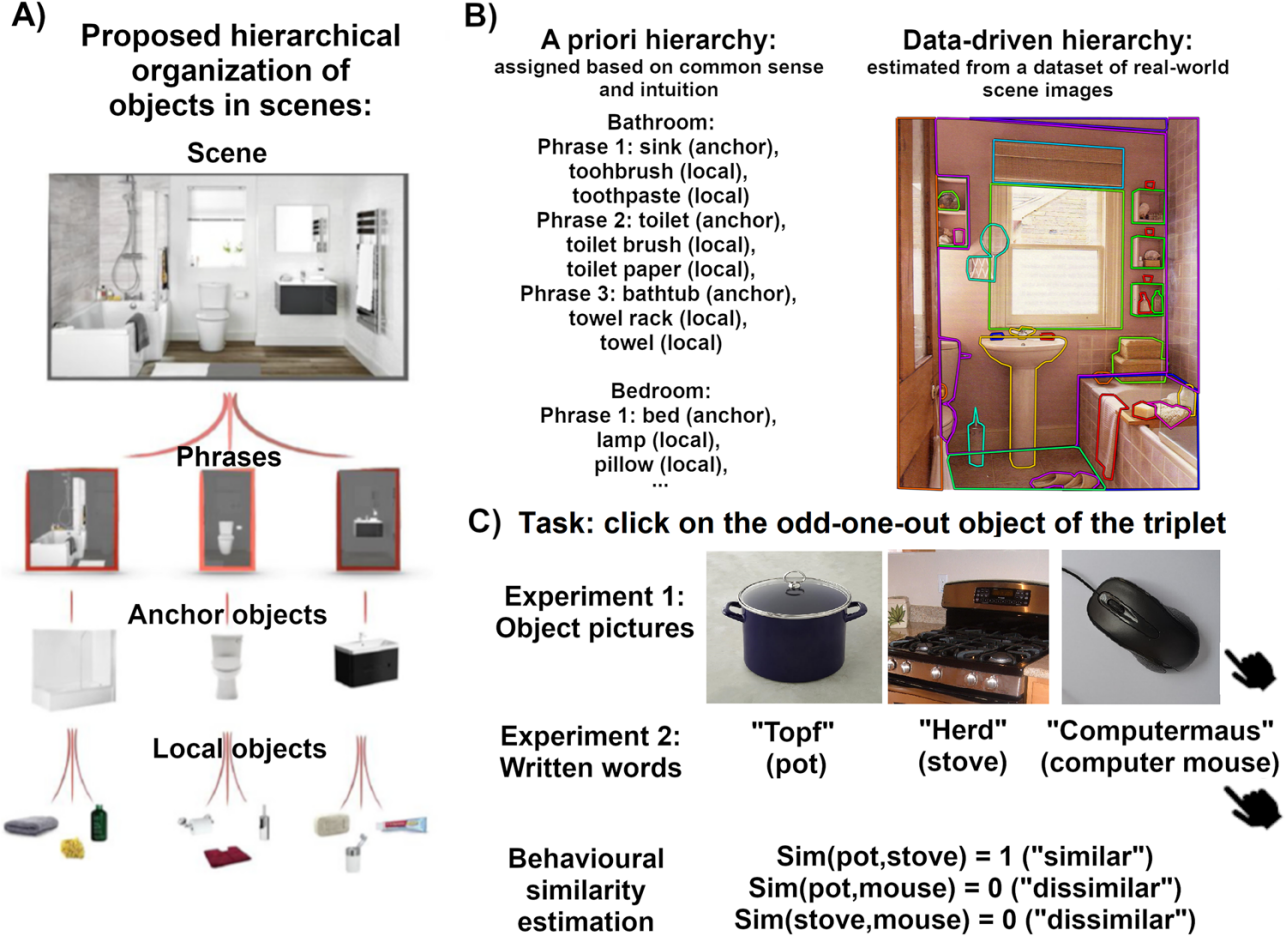
(**A**) Schema of the hierarchical structure of objects in scenes tested in the study: a scene is divided into clusters (phrases) and each phrase is formed by one anchor objects and several local objects (figure adapted from^8^); (**B**) Estimation of hierarchical measures using a priori assignment of objects to a scene, phrase and object type or using a datasets of annotated and segmented images from which we can extract co-occurrence and clustering information (image taken from the dataset^28^ and visualized through LabelMe^29^); (**C**) Example of a trial from Experiment 1 and Experiment 2 showing a triplet of objects (pictures or words), as well as the way we measured behavioural similarity from the response in the trial: pairs including the selected “odd-one” object have minimal similarity while the pair including the unselected objects has maximal similarity. Object images are taken from^30^ and are not the one used in the real experiment.

More recently, it has been proposed that Scene Grammar could be structured according to a hierarchy^8^: a scene on the top level is divided into meaningful clusters of spatially related objects, which we refer to as “phrases”; in every phrase, one object holds a special status (“anchor object”), with strong predictions regarding both the identity and position of the other objects within the cluster (“local objects”; Fig. 1A). Anchor objects are proposed to be typical (i.e., frequently present) of a scene, bigger in size and rather stationary (e.g., a sink), while local objects tend to be smaller and more moveable (a toothbrush). The proposed role of this hierarchy entails that during complex behavior within a scene, like object search or interaction, we first and foremost process objects based on their phrasal membership within a scene.

So far, mostly the top “scene level” as organizing structure of objects has been investigated. It is believed that priors regarding object-to-object and object-to-scene relationships are activated after a quick extraction of a scene’s “gist”^9,10^. As a result, typically studies have manipulated the consistency between an object and its background scene (e.g., a priest in a church vs. a football court^11^), and have tried to identify which ingredients of a scene are sufficient to retrieve this contextual knowledge (e.g., color and texture^12^; orientation^13^; materials^14^; layout^15^; for a review^16^).

The “phrase level” has hardly received any attention thus far, but there have been attempts to disentangle what the role of pairs and groups of objects is in supporting object identification. For instance, co-occurrence (a pot and a stove) and spatial dependency (a pot on top of a stove) between objects have been also found to be relevant for object processing during visual search^17,18^ and object recognition^19,20^, even beyond the effect of background scene information^21^. Indeed, the complex network of object-to-object relationships seems to be retrieved even when objects are seen in isolation on a neutral background, as shown by the correlation between fMRI patterns evoked by single object pictures and a computational model that uses distributional statistics of objects in scenes^22^. Besides, typical semantic and spatial arrangements of multiple objects are processed in a more efficient way both at behavioral and neural level^23,24^ supposedly due to a grouping mechanism that allows to reduce the complexity of visual input. This grouping based on meaning and spatial relationship might also be supportive of extraction of action affordances, which seems to play an important role in scene understanding^25^ and might be the organizing principle behind the phrasal structure in man-made scenes^2^.

Finally, for what concerns the “object type level”, first empirical results supporting the prominent role of anchor objects in structuring a scene came from a study where participants were asked to arrange objects in a virtual environment according to their scene grammar (creating a typical arrangement of objects in scenes^7^): Anchor objects were preferentially used during initial stages of object arrangements underlining their role as primary building blocks of a scene. The important role of anchor objects in visual search has been further corroborated by a series of eye-tracking experiments where the absence of anchor objects (e.g., the toilet being replaced by a washing machine) resulted in less efficient search performance as seen in faster RTs and reduced gaze coverage of the scene^26^. These results were then replicated in more ecologically valid and immersive setting provided by virtual reality (VR^27^). Participants had to search for target local objects within virtual environments that either displayed anchor objects or anchors replaced by gray cuboids in the same position. The presence of anchors had strong beneficial effects on search behavior as seen in more efficient gaze and body movements.

The goal of the current study was to investigate whether the contextual knowledge associated with mental representations of object is organized according to a hierarchy, where the levels of scene, phrase, and object type (anchor vs. local) can be distinguished. Moreover, we wanted to assess whether the organization of object representations is modality-specific or independent of specific modalities (e.g., verbal and non-verbal stimuli^31^).

**Figure 2:**
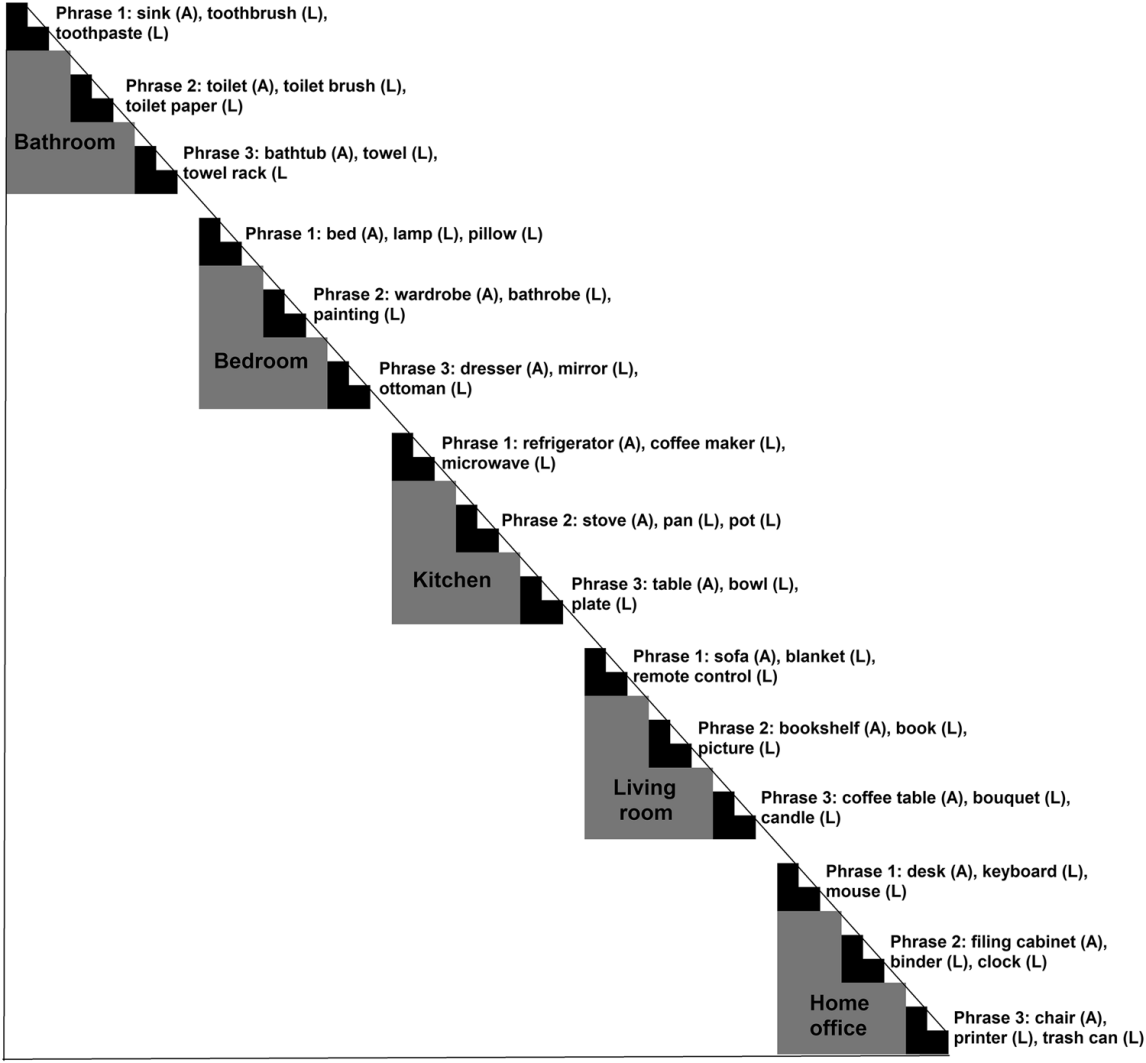
One half of a symmetric Representational Dissimilarity Matrix (RDM) showing the organization of individual object pairs based on the a priori hierarchical organization. Gray and black portions of the triangle represent pairs of objects assigned to the same scene category, while black portions represent pairs of objects assigned to the same phrase within the scene. Scene category labels and composition of the phrases are also reported, the letter (A) indicates an anchor object, the letter (L) indicates local objects. The remaining white portion of the triangle represents pairs of objects that are assigned to different scenes. This order of objects is maintained in the RDMs and used to represent different levels of the hierarchical models (see Fig. 3).

**Figure 3:**
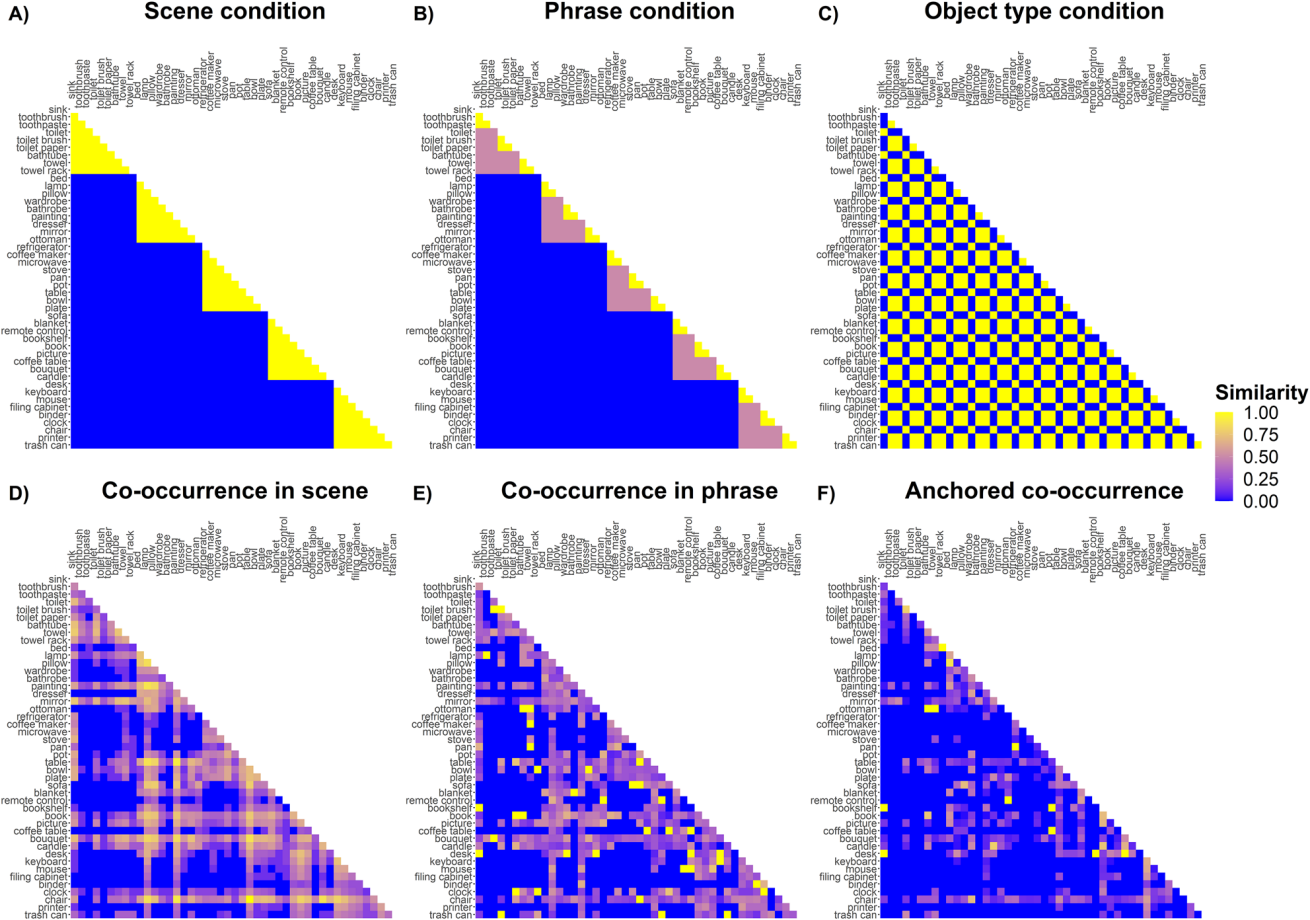
Representational (Dis)similarity matrices (RDMs) for the a priori hierarchical predictors (**A**–**C**) and for the data-driven hierarchical predictors (**D**–**F**). RDMs are symmetric matrices where entries on rows and columns are the objects stimuli, and cells represent pairwise similarity along a specific dimension. In (**A**–**C**), yellow represents pairs of objects that are assigned to the same scene, phrase or type (maximal similarity), while blue represents pairs that are assigned to different scenes, phrases or types (minimal similarity). In (**D**), the log10(counts + 1) of co-occurrence in scene is normalized to span between 0 (blue, few counts) to 1 (yellow, many counts). In (**E**) and (**F**), the colors represent proportion of counts to the total co-occurrence counts of each pair.

To achieve these goals, we organized a set of everyday objects according to the above-mentioned hierarchical structure in two ways (Fig. 1B): one based on common-sense and intuition (a priori hierarchy model), and the other one based on the distribution of objects in a real-world image dataset^28^ (data-driven hierarchy model), both organizing objects on three levels: scene, phrases and object types. Then, we collected pairwise similarity ratings for the set of objects, adapting an “odd-one-out” triplet task (Fig. 1C) previously used to study perceptual and conceptual dimensions underlying mental representation of objects^32^. Finally, we compared the odd-one-out ratings to the hierarchy models using Representational Similarity Analysis (RSA^33^), which allows to estimate if the representational space underlying behavioural responses is structured according to the levels of our proposed hierarchical organization, representing pairwise similarity of both behaviour and hierarchical models in terms of Representational (Dis)similarity Matrices (RDMs; see Fig. 2 for the organization of individual objects in the RDMs, and Fig. 3 for RDMs of each hierarchical predictor). To estimate the simultaneous impact of different levels of the hierarchy and different types of hierarchy, we combined RSA with Generalized Linear Mixed-effects Models (GLMMs^34^).

## Results

**Figure 4:**
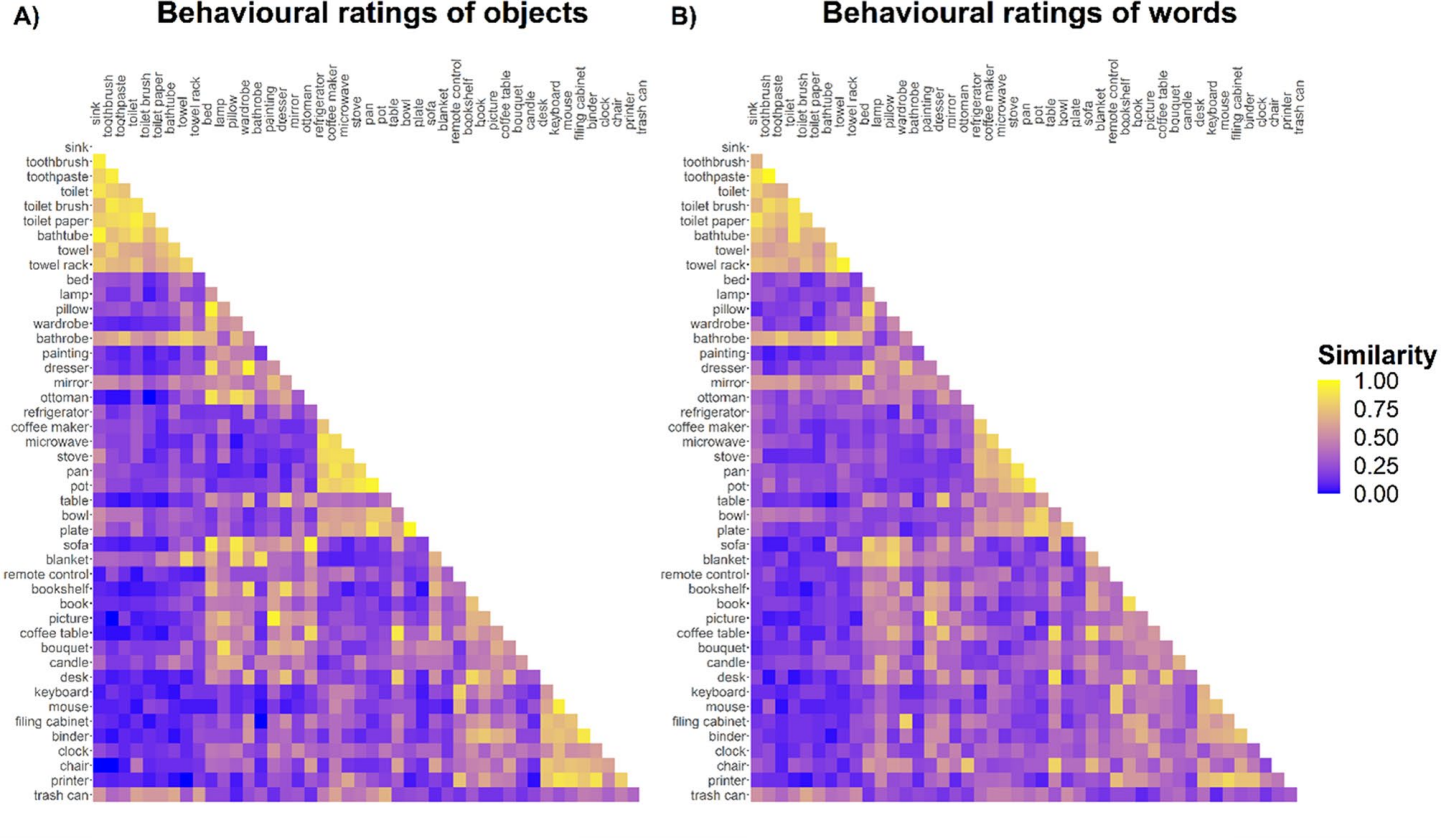
Representational (dis)similarity matrices (RDMs) for the ratings collected in Exp 1 (object pictures, **A**) and Exp 2 (words, **B**). Cells represent pairwise similarity ratings averaged across all the triplets where the pair was present. Every pair was presented in a triplet with all the other remaining objects (“context object”), and it was judged either as similar (1) or dissimilar (0), so that in the RDMs pairwise similarity spans from 0 (never judged as similar) to 1 (always judged as similar).

Ratings divided by modality were plotted in the RDM format (Fig. 4), where every cell represents the pairwise similarity ratings for a given pair averaged across all the triplets where the pair is present. The GLMM resulted to be singular, due to the random factor term (1 | *participants*) explaining no variance, since this was already explained by the other two random factors (1 | *pairs*) and (1 | *context objects*), that identify unique observations.

To evaluate potential multicollinearity in the model, we computed the variance inflation factors (VIFs) for each term in the model, using the *check_collinearity* function in R (package “performance”^35^). Typically, when VIFs are below 5, there is low correlations between predictors and the model does not need any adjustment, as it was in our case (VIFs and correlations among predictors are shown in detail in Supplementary Material 1).

**Figure 5:**
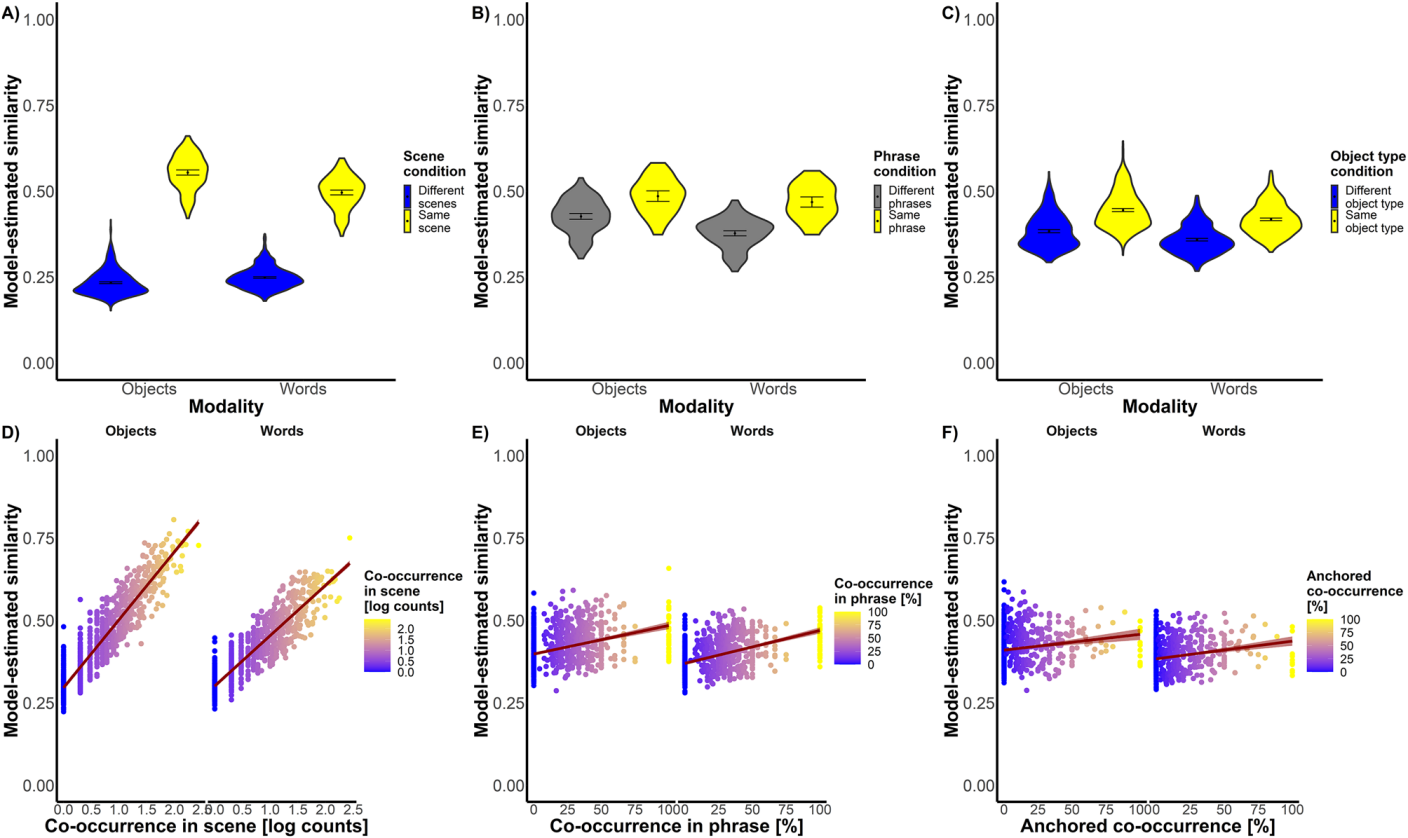
Model-estimated effects of the hierarchy predictors on pairwise similarity ratings for object pictures and words. Colours of violins and points reflect the values of pairs for the given predictor and match the ones in the RDMs showed above. Stimulus modality is indicated by x-axis position (left = objects, right = words). Points and violins reflect estimated similarity for each pair of objects averaged across all the different contexts (i.e., the third object a triplet) in which they were presented. 95% confidence interval are represented by error bars in the violins (point is the mean), and by the shaded area around lines for continuous predictors.

Results from the GLMM (Fig. 5) showed a main effect of stimulus modality (β = − 0.107, SE = 0.031, z = − 3.448, p = 0.001), with objects pictures estimated to be more similar to each other than object words. The a priori hierarchical structure was reflected in participants’ similarity ratings, with significant main effects of scene condition (β = 1.078, SE = 0.075, z = 14.474, p < 0.001), phrase condition (β = 0.270, SE = 0.128, z = 2.111, p = 0.035), and object type condition (β = 0.245, SE = 0.048, z = 5.106, p < 0.001), showing that objects belonging to the same scene/phrase/object type were considered more similar than objects belonging to different scenes/phrase/object types. At the same time, we also found main effects of the data-driven hierarchy predictors measuring co-occurrence in scene (β = 0.397, SE = 0.029, z = 13.922, p < 0.001) and co-occurrence in phrase (β = 0.063, SE = 0.028, z = − 2.229, p = 0.022), where in both cases the more two objects co-occurred, the more they were judged to be similar. However, the anchored co-occurrence between two objects was not significantly reflected in pairwise similarity ratings (β = 0.005, SE = 0.028, z = 0.165, p = 0.869). Overall, these results already show a hierarchical organization of mental representations not only on the scene level, but also at the phrasal and object type level.

Regarding the covariate measures (see Supplementary Material 2), we found main effects of the early layer of AlexNet DNN (β = − 0.133, SE = 0.025, z = − 5.317, p < 0.001), with pairs that looked more similar in terms of low-level visual features being considered less similar at behavioural level, while the main effect of late layer of AlexNet (β = 0.126, SE = 0.031, z = 4.078, p < 0.001) showed that object pairs that looked more similar in terms of high-level visual features were also estimated to be more similar by our participants. Finally, we detected a main effect of word embeddings (β = 0.338, SE = 0.025, z = 13.363, p < 0.001), with object pairs that have stronger similarity in terms of distributional semantics features being considered more similar. These results show that distinction emerging from both complex visual features (AlexNet late layer) and word meaning (Word embeddings) are important factor in determining the mental representation supporting behaviour, while contrary to that, similarity based on low-level visual features (AlexNet early layer) acts as a confound making more similar objects less distinguishable.

In terms of interaction between stimulus modality and our predictors, the model showed a significant effect in scene condition (a priori predictor, β = − 0.280, SE = 0.050, z = − 5.601, p < 0.001), and in co-occurrence in scene (data-driven predictor, β = − 0.124, SE = 0.019, z = − 6.361, p < 0.001), where in both cases the effect of the hierarchical predictor was found to be stronger in ratings of object pictures than ratings of words. Object ratings had also stronger effect of the late layer of AlexNet than word ratings (β = − 0.112, SE = 0.022, z = − 5.157, p < 0.001), while word ratings had a stronger effect of word length than object ratings (β = 0.082, SE = 0.028, z = 2.977, p = 0.003; for more details, see Supplementary Material 2). This is expected since both predictors are estimated based on their preferential stimulus modalities (AlexNet activation with object pictures; Word length with words), and signifies that these dimensions are more strongly related to modality specific representations compared to the hierarchical predictors.

For more details regarding how object size, manipulability and moveability interact with different object types (anchor and local objects) see Supplementary Materials 3 and 4.

## Discussion

Objects in visual scenes are arranged in a structured way. These structural regularities are learnt and stored in long-term memory (“scene grammar”) to make meaningful predictions and efficiently perceive and interact with the environment^2^. In this study, we wanted to explore whether scene grammar is organized in a hierarchical way. We hypothesized that at the top of the hierarchy, objects are grouped together according to whether they appear in the same context (scene level), followed by objects that spatially cluster within that context (phrase level), which again consist of anchor objects that hold strong predictions about identity and position of other local objects within a cluster^8^. Moreover, we wanted to understand if this organization emerges differently in one modality than the other (e.g., object pictures vs. written words). For this purpose, we adopted the odd-one-out task as introduced by Hebart and colleagues^32^, a method that has been used to study perceptual and conceptual dimensions underlying mental representation of objects.

We have shown that when participants are asked to judge the similarity between pairs of objects, the underlying mental representations seem to be organized according to our proposed hierarchy. That is, pairs of objects that were assigned a priori to the same scene, to the same phrase, or to the same object type, were judged as more similar than pairs of different scenes, phrases and types. This finding largely held up even when the hierarchy was estimated from statistical distributions of objects in real-world images^28^. Besides, we showed that these results were overall consistent and stable across modalities, with only the scene level predictors showing an even stronger effect for object pictures than words. Finally, we highlighted how the a priori division of objects between anchors and local objects is strongly based on object size and moveability, as previously proposed and showed^26^.

To our knowledge, this is the first attempt to explore whether the hierarchical organization of objects in scenes is incorporated into our mental representations. Previous research either focused on effects of scene context on object processing (e.g.^2^; for a review see^16^) or on the relationship between anchors and related local objects (e.g.^26,27^). Here, we aimed at bridging the gap between these two levels considering the role of meaningful clusters of objects (“phrase” level) as an intermediate structure within the hierarchy.

Employing two different sources of estimation of the hierarchy allowed us to draw some interesting conclusions. The weak correlations between a priori and data-driven hierarchy predictors and the absence of multicollinearity (see Supplementary Material 1) show that, despite the same direction of the effects, the two models of hierarchy are only partly overlapping. We can only speculate about the reasons of these differences, which might also speak to the limitations of both types of hierarchy estimations: on the one hand, previous research has shown that subjective experience of how frequently objects in the world occur is overestimated^36^, which might have resulted in differences between a priori estimations and measures taken from the distribution of objects in labeled image databases; on the other hand, it is important to note that any given dataset of annotated images only represents a rough (and often biased) approximation of the real-world distribution of objects. Compared to word frequency measures based on corpora of at least 20 million words^37^, fully annotated image datasets are much smaller in size (in our case, circa 45,000 annotations). The two hierarchical organizations (a priori vs. data-driven) might also reflect object processing in two different ways: for instance, the a priori hierarchy is based on discrete, dichotomic divisions of objects dependent on whether they appear in the same context or not, and therefore might be used when a task requires the processing of rough contextual information; on the other hand, the continuous co-occurrence measures from the data-driven approach might offer a more fine-grained representation of object-to-object contextual information when necessary. Using distributional properties of objects in scenes as calculated from annotated datasets (similar to research on language) is becoming increasingly popular and provides interesting insights on learning statistical regularities in both vision an in language^22,38^, offering an alternative to traditionally employed categorical divisions based on experimenters’ intuition or crowd-sourced ratings.

The measures that can be extracted from this type of datasets can offer even more fine-grained information than what we highlighted here: for example Boettcher et al.^26^ measured that the relationship between anchor and local objects has strong regularities on the vertical axis, that is, it is possible to predict the position of a certain local object from a certain anchor object in terms of “is above” or “is below”, but not as much on the horizontal axis (“is left of” or “is right of”), similar to linguistic grammar where in most languages the components of a phrase (e.g., subject and object) have predictable positions with respect to each other. This seems to match the intuition that the structure of a room is much more vertically organized: objects typically found on the lower part of a room tend to differ from objects typically found in the top part of the room (e.g., shoes usually are found on the floor, while paintings are hanging up on the wall), while on the horizontal axis there is much more variability (e.g., the towels can be found either left or right of the shower. This vertical organization of the environment seems to indeed also be reflected in the neural representation of scenes^39^.

The significant results of both types of hierarchy predictors suggest that, despite some of their limitations, these are capturing aspects of the visual world that seem to be incorporated in our mental representations of objects. This is particularly interesting as these layered representations seem to be triggered by simply viewing isolated objects or words. It is important to point out that—similar to Hebart and colleagues^32^—no explicit definition of similarity or specific instructions on how to judge the (dis)similarity of the three presented objects/words were given to the participants when performing the “odd-one-out” triplet task. The aim was to collect similarity judgements that are not biased towards specific dimensions while allowing different dimensions to emerge in different contexts. For example, “cat” and “elephant” might be similar in a triplet with “table”, based on animacy, but “cat” and “elephant” might be dissimilar in a triplet containing “dog”, where the similarity might be based on whether the animals are pets or not. However, it has been shown that—using the same triplet task with different similarity instructions—it is possible to measure the flexibility of mental representations in highlighting one dimension more than others according to task demands^40^. We believe this could also apply to the hierarchical organization of objects in scenes, whose strength in shaping mental representation might be increased by tasks that require interactions with objects (e.g., judging similarity based on function) and reduced by tasks that rely less on object-to-object contextual relations (e.g., judging similarity based on visual features). Future investigations directly comparing different “odd-one-out” triplet tasks might shed more light on these aspects.

A question that remains open is whether this hierarchical organization is present in every type of scenes. In the present study, we have employed only an organization that relates to indoor man-made environments, because we believe that here the hierarchical structure is optimized to efficiently perform everyday actions like brushing teeth or cooking. Outdoor scenes in general, and natural scenes in particular, might show less of a hierarchical structure. First of all, in the way they are experimentally investigated, they have much bigger scale than indoor environments. This has consequences on navigational and action patterns, which differs from the ones of smaller scale indoor scenes. Second, natural scenes, in which man-made objects are rare or even absent, lack object arrangements that reflect the need for efficient human-object interaction. That said, nature of course has its own “grammar” as well (e.g., the way that rivers flow or rocks fall into place), and it might be worth investigating the hierarchical structure of natural scenes and how these might be mirrored in mental representations.

While we did not measure brain responses in this study, it is still worth discussing how such hierarchical organization could be implemented in the brain. For instance, the hierarchical organization of objects in scenes might be represented in the parahippocampal cortex (PHC), in the anterior part of the ventral-temporal cortex. Within the PHC lies the parahippocampal place area (PPA), a scene-selective region which shows stronger activation for scene stimuli rather than single objects^41^. Subsequent investigations have suggested that PPA/PHC might represent spatial and non-spatial context in a more general way^9,42^, and not just based on visual scenes. This is in line with recent findings that viewing single isolated objects evoked a complex representation of objects’ co-occurrence in the anterior portion of PPA^22^. Here also lies the perirhinal cortex, which has been proposed to represent semantic information for individual objects^43^, and is the medial portion of the Anterior Temporal Lobe (ATL), which has been proposed to be the primary hub of the semantic network^44^.

Finally, our results—according to which hierarchical predictors show significant main effects and minor differences between modalities—suggest that scene grammar might act on domain-general representations. That is, the hierarchical structure of our visual world might be incorporated into semantic memory representations of objects which are accessed when an object’s meaning is retrieved from processing input from different modalities, here either pictures or words. Some visual and hierarchical features are not completely independent, but we took great care to not have extreme levels of multicollinearity invalidate the interpretation of our results (see Supplementary Materials for correlation plots and VIF estimates). We therefore want to propose that a scene’s hierarchical structure is incorporated into the abstract semantic representations of both objects and words that can be used to flexibly form predictions when encountering new visual environments or written text. We believe that with this paper we were able to demonstrate that using several visual and linguistic covariates, as well as measuring effects on both object pictures and words, we can now provide some first evidence that the hierarchical predictors are (1) independent of the visual and linguistic dimensions measured here and (2) are independent of the specific modality of stimulus presentation.

To conclude, in the current study we provided first evidence that abstract mental representations of objects in scenes might be hierarchically organized, incorporating not only scene semantic information at the highest level, but also a more fine-grained, mid-level phrasal structure, as well as distinctions of object types. We therefore believe that these phrasal substructures of scenes play an important role in the organization of our mental representations of the world and therefore should be considered when studying visual cognition.

## Materials and methods

### Participants

Eighty-six participants took part in our study. Half of them took part in Experiment 1 (age: M = 24.72 years, SD = 5.33 years, range = 18–40 years; gender: F = 31, M = 12), the other half took part in Experiment 2 (age: M = 22.60 years, SD = 5.18 years, range = 19–50 years, 1 person did not report age; gender: F = 28, M = 15). The number of participants in each experiment (N = 43) was determined as the optimal ratio between the total number of unique trials and an optimal number of trials to present to a single participant. All participants reported that they had normal or corrected to normal vision and had no history of psychiatric or neurological disorders. Participants of Experiment 2 also reported to be German native speakers. Additionally, a third group of participants (N = 20), who did not take part in either Experiment 1 and Experiment 2, participated in a rating experiment to judge some features of objects (age: M = 22.9 years, SD = 4.00 years, range = 19–35 years; gender = 12 F, 7 M and 1 NB). These participants matched the same criteria of participants in Experiment 1. No minors participated in the study. All participants gave their informed consent and received course credits or monetary reimbursement for their participation. The Ethics Committee of the Goethe University Frankfurt approved all experimental procedures (approval # 2014-106), that have been performed in accordance with the Declaration of Helsinki.

### Stimuli

Forty-five everyday indoor object concepts were selected for the study (see section below for more details). For Experiment 1, pictures of the objects in isolation were downloaded from copyright-free internet databases (e.g., https://pnghunter.com/, http://pngimg.com/, https://www.cleanpng.com/), pasted on a white background, grey-scaled to rule out influence of color, and resized to 392 × 392 pixels (jpg format). For Experiment 2, we used the German words associated with the objects, presenting them in bold black Arial font, with the first letter in uppercase and the other letters in lowercase, as by correct German spelling for nouns.

### Measures of scene hierarchy

To predict similarity judgments as a function of scene hierarchy, we estimated two sets of scene hierarchy measures.

- * A priori hierarchy measures*: these measures were based on intuition of experimenters as well as common sense; therefore, we selected our 45 stimuli as typically belonging to one of 5 different indoor *scenes* (bathroom, bedroom, kitchen, living room and home office). For every scene, we divided objects in 3 *phrases*; within every phrase, 1 object was identified as *anchor object*, and the other 2 as *local objects* (Figs. 1B and 2).
- *Data-driven hierarchy measures*: these measures were based on a dataset of real-world scene images containing pixel-wise segmentation and annotation of objects^28^. The dataset contained 3499 unique coloured images, grouped into 16 scene categories (both indoor and outdoor, natural and man-made, and including the 5 categories considered in the a priori assignment), with more than 48,000 annotations grouped into 617 different object categories (including the 45 objects selected for the study). Annotations were done by 4 different workers using the LabelMe tool^29^ and were carefully cleaned of misspelling and synonyms (Fig. 1B).

Following the procedure used in Boettcher et al.^26^, we first pre-processed the annotation and segmentation data in MATLAB (MathWorks, 2018), extracting identity, coordinates and centroids of each object in the 2D space of pixels of each image. Further analysis were carried on in R (version 3.6.3, R Core Team, 2020). Second, we discarded objects that have a more structural function (e.g., walls, windows, ceiling, doors, pipes) rather than being relevant for the object-to-object relationship we were interested in investigating, leaving us with 567 unique object categories. Given the structure of the data, we could compute how many times *two objects co-occur in the same image*, which is the data-driven counterpart of the *scene level* of the hierarchy. Then, representing the objects in an image through their centroids and the image area as a 2D space, we ran a clustering algorithm to find the optimal spatial grouping of objects in every scene: the algorithm was based on the partitioning around medoids clustering method and estimated the number of clusters using average silhouette width (*pamk* function from R package “fpc”^45^). We identified the resulting *clusters of objects as phrases*, and within every cluster, we identified the *object with the largest area as anchor object*, while the other objects in each cluster were considered *local objects*.

### Visual and linguistic covariates

Additionally, to ensure that effects of the scene hierarchy did not emerge from a confound of lower-level information, we estimated several measures of visual features (for object pictures in Experiment 1) and linguistic features (for words in Experiment 2):

- *Visual measures* (for pictures): we estimated visual features of our object images feeding them to a pre-trained Deep Neural Network (DNN), a state-of-the-art computer vision algorithm that is trained to perform object categorization at human-like level. In our case, we used the popular AlexNet, trained on the ImageNet dataset^46^. AlexNet, like most DNNs, is based on many sequential layers of processing units, which extract and transform features from the previous layer. The first layer extracts features from the input layer, which is formed by the pixel values of an image; then the information is transformed in an increasingly complex way through the many intermediate layers until it reaches the final output layer, which assigns the image to one category (e.g., “cat”). We estimated unit activations for our object images in 3 different layers of AlexNet: convolutional layer 1 (*conv1*, “*early layer*”), which processes low-level visual features (e.g., edges, brightness); convolutional layer 4 (*conv4*, “*mid layer*”), which process mid-level visual features (e.g., shape); and the fully connected layer 7 (*fc7*, “*late layer*”), which processes high-level visual features (complex configurations, like faces, handles, etc.).
- *Orthographic measures* (for words): we estimated orthography of our word stimuli using 2 measures: *word length*, as the number of letters in a word; *orthographic distance* from neighboring words (i.e., words that differ for a letter from a target word), computed using the OLD20 measure^47^.
- *Distributional semantic measures* (for words): distributional semantic is a model of word meaning based on the idea that words that appear in similar linguistic contexts (i.e., they have a similar distribution in text) have similar meaning (for a review^48^). This approach has been widely used in Natural Language Processing (NLP) to create algorithms that use distributional measures from text corpora to build representations of word meaning and perform operations on it. One common way of representing word meaning in NLP is through *Word embeddings* which are multi-dimensional vectors. Words whose embeddings are closer in this vector space have also similar meanings. For our set of word stimuli, we used the embeddings trained on German Wikipedia using fastText and the skip-gram model with default parameters^49^.

### Object features

To better understand what features underly the division of objects between anchors and local objects, we have collected ratings about three dimensions that have been discussed in connection to the status of anchor and local objects: *real-world size* (how big an object is), *moveability* (how easily an object is moved in space) and *manipulability* (how much the position of an object or of one of its part or its configuration is changed during the interaction with it).

### Apparatus and procedure

Apparatus and procedure were mostly identical across Experiments 1 and 2. Where there were differences, those are reported explicitly. For the study, we adapted an “odd-one-out” triplet task introduced by Hebart and colleagues, which elegantly is used to collect pairwise similarity judgments of object pictures^32^. First, we generated all the possible combinations of triplets of stimuli (45!/(3! × (45 − 3)!) = 14,190 unique triplets). We then divided the triplets randomly into 43 groups of 330 triplets, to have a practical number of trials and participants. Every participant, therefore, performed the task on a different subset of triplets.

Experiments were programmed in Python using PsychoPy (version 2020.2.4, Builder GUI^50^) and administered online through the hosting platform Pavlovia (https://pavlovia.org/). Participants were asked to start the experiment only when they had between 30 min/1 h of free time and only when they could carry on the procedure with calm and in an undisturbed environment. Instructions told participants they would have seen triplets of stimuli and their task would have been to choose the “odd-one-out” stimulus, i.e., the one they considered the least similar to the other two. No explicit definition of similarity was given to participants, as in the original study. This is in line with the purpose played by the “odd-one-out” triplet task: similarity between a pair of objects is evaluated across multiple trials (i.e., triplets), in which the context keeps varying (i.e., the third object of the triplet). This way, many different dimensions are allowed to emerge and be prioritized to judge the pair similarity, giving back a more complex picture of object representations^32^.

In our study, triplets were presented on a white background screen, with one stimulus on the left, one stimulus in the center and one stimulus on the right (the position of every stimulus in the triplet was randomized within every triplet before the presentation; Fig. 1C). Experiments were programmed so that stimulus size were normalized based on screen size, so that every participant saw stimuli occupying the same proportion of screen: each picture spanned about 1/4 of width and height size, while each word spanned about 1/10 of height size and varying width size according to word length. To choose the odd-one-out stimulus, participants had to press the corresponding arrow (left arrow for the stimulus on the left, down arrow for the stimulus in the center, right arrow for the stimulus on the right). Once they pressed the key, a 500 ms black fixation crossed appeared in the center of the screen and then the next triplet was presented. Trials were divided into 6 blocks, between which participants could take a break. Participants were allowed to take as much time as they wanted to make their “odd-one-out” decision, and if they could not recognize one of the stimuli, they were asked to make their decision based on what they thought the stimuli were.

In the object features rating experiment, participants performed the ratings of moveability, manipulability, and real-world size in three different blocks (in this order). Within every block, participants saw the pictures of the object stimuli from Experiment 1 one at the time (in randomized order), together with the rating question (above the picture) and a 6-point likert scale (below the picture). Before the block, they were presented with a definition of the investigated dimension, and were asked to press a number between 1 to 6 corresponding to their judgments.

### Analysis

To analyze how measures of scene hierarchy predict pairwise similarity judgments, we combined two main analytical approaches: Representational Similarity Analysis (RSA^33^) and Generalized Linear Mixed-effects Models (GLMMs^34^). RSA is a tool that allows comparison of different sources of data that have different dimensionalities (brain data, behavioral data, computational models, stimulus features). To do so, it requires the creation of Representational (Dis)similarity Matrices (RDMs), which are symmetric matrices where column and row entries are typically corresponding to the different stimuli (Figs. 2, 3). Every cell in an RDM contains a measure of (dis)similarity for that pair of stimuli. Once the different sources of data are represented in the same RDM format, it is possible to compare them and estimate how similar two RDMs are, i.e., how the structure of pairwise similarity in one source (e.g., behavior) is predicted by the structure of pairwise similarity in another source (e.g., a computational model).

In our study, we followed this approach to compute pairwise similarities from the “odd-one-out” triplet behavioral task, as well as from the measures of hierarchy and covariates introduced above.

- *Behavioral similarity*: we estimated behavioral similarity between pairs of stimuli in a dichotomic way: similar (dummy coded as 1) vs dissimilar (dummy coded as 0). This estimate was assigned as a result of the “odd-one-out” choice on every triplet. Given a triplet (e.g., A, B and C), once an “odd-one” stimulus is selected (e.g., C), the similarity between the unselected stimuli results to be maximal (Sim(A,B)  = 1 → “similar”), while the similarity between the “odd-one” stimulus and one of the unselected stimuli results to be minimal (Sim(C,A) = 0 → “dissimilar”; Sim(C,B) = 0 →  “dissimilar”; Fig. 1C).
- * A priori hierarchy similarity*: we estimated pairwise similarity based on the hierarchy status assigned a priori. This results in 3 categorical predictors. First, we considered *scene condition*, with dichotomic categorization: pairs from the same scene (dummy coded as 1) vs pairs from different scene (dummy coded as 0). Then, we considered *phrase condition*, with three groups: pairs from the same phrase (1) vs pairs from different phrases within the same scene (0.5) vs pairs from different phrases in different scenes (0). Finally, we considered *object type condition*, with two categories: pairs of objects of the same type (1) vs pairs of objects of different type (0), where object type refers to the object being either an anchor object or a local object.
- *Data-driven hierarchy similarity*: we estimated pairwise similarity based on the hierarchical status emerging from the clustering procedure on the labelled image dataset. This results in 3 continuous predictors. First, we estimated a measure of *co-occurrence of pairs in a scene*, as the number of times a pair appears in the same image; in the analysis we used log10 (counts + 1), so that we had a more uniform distribution along this dimension and avoid having -Infinite values. Then, we estimated a measure of *co-occurrence of pairs in a phrase*, as the proportion of co-occurrence counts where a pair not only appears in the same image but also in the same cluster. Finally, we estimated a measure of *anchored co-occurrence*, as the proportion of co-occurrence counts where one object of a pair is “anchored” to the other.
- *Covariates*: for the visual, orthographic, and distributional semantic measures, similarity was estimated in different ways. For multidimensional measures (i.e., the 3 AlexNet layers and the Word embedding), similarity was estimated by computing the product–moment correlation coefficient between pairs of vectors (e.g., the embedding vector for “pan” and the embedding vector for “pot”); for mono-dimensional measures (i.e., word length and orthographic distance), similarity was computed as the absolute value of the difference between the two values of each pair (e.g., the absolute value of the difference between word length for “pot” and word length for “pan”).

GLMMs are an extension of Linear Mixed-effects Models (LMMs^51^) for responses/dependent variables that have a non-gaussian distribution (in our case, the bimodal dichotomic behavioral similarity). The main advantage of (G)LMMs over simple regression models and ANOVAs is that one can consider each trial from each participant simultaneously, without the need for aggregation or separate estimation of the effects across participants and item (i.e., crossed random effects of items and participants^52^). Therefore, the response is estimated based on several predictors (fixed factors) and considering grouping factors that have common portion of variance (random factors). Using R syntax, our model had this structure:

$$behavioral\;similarity\sim stimulus\;modality \times (scene\;condition + phrase\;condition + object\;type\;condition + cooccurrence\;in\;scene + cooccurrence\;in\;phrase + anchored\;cooccurrence + covariates) + (1|participants) + (1|pairs) + (1|context\;objects)$$

In the formula, on the left of the tilde (~), we have the response, i.e., the dichotomic behavioral similarity from the triplet task; on the right of the tilde, we have the predictors, i.e., the categorical and continuous pair similarity from the a priori and data-driven hierarchical organization, as well as pair similarity for covariate measures; finally, we have the random factors, i.e., participant, pair, and context object (the third object in the triplet). We fitted the statistical models via maximum likelihood estimation, and continuous predictors were scaled, as this typically improves model fit. For categorical predictors, we planned specific contrasts between conditions: for scene condition, the contrast was set to *same scene − different scenes*; for object type condition, the contrast was set to *same object type − different object types*; for phrase condition, one contrast was set to *same phrase − different phrases of the same scene*, while the other contrast was set to (*same phrase* and *different phrases of the same scene*)* − different phrases of different scenes*. Since this last contrast is identical to *same scene − different scenes*, and since the scene similarity and phrase similarity predictors are highly correlated, we removed from the model the *scene condition* predictor and incorporate its contrast in the *phrase similarity* predictor. This way, we removed redundancies and reduced multi-collinearity to an acceptable level. Besides, every measure was put in interaction with the categorical predictor *stimulus modality*, which compares the effect of the measures between words and objects pictures. Finally, for random effects, we included only an intercept term, so that we followed the recommendations of Bates et al. about parsimony in random effect structure^53^.

RSA was previously used in combination with general linear model (e.g.^39,54^), modeling response RDMs of different participants (from brain or behaviour) as a linear combination of multiple predictors RDMs (from stimulus features or computational models) and going beyond the simple 1-to-1 correlation between response and predictor RDMs originally presented in RSA. In our approach we went one step further: since the similarity of each pair is estimated multiple times in different context (the third object of the triplet), and since each context object appeared multiple times with different pairs, we considered these additional sources of random variance (pairs and context objects) exploiting the flexibility of GLMMs.

Analysis was performed using R (version 3.6.3, R Core Team, 2020).

## Supplementary Information

Supplementary Information.

## Data Availability

Data and scripts are available at the following link: https://osf.io/tx4m5/.
