# Supplementary material for: Hierarchical organization of objects in scenes is reflected in mental representations of objects
Source: Sci Rep. 2022 Nov 23;12:20068. doi: 10.1038/s41598-022-24505-x (PMC9684142; doi:10.1038/s41598-022-24505-x)
Supplement: Supplementary file 1 — Supplementary Information. [file 41598_2022_24505_MOESM1_ESM.docx]

**Supplementary Materials**

**Supplementary Materials 1 – Factor correlations and VIFs in the main model**

**Sup. Fig. 1 –** Matrix of correlations between the predictors used in the model

**
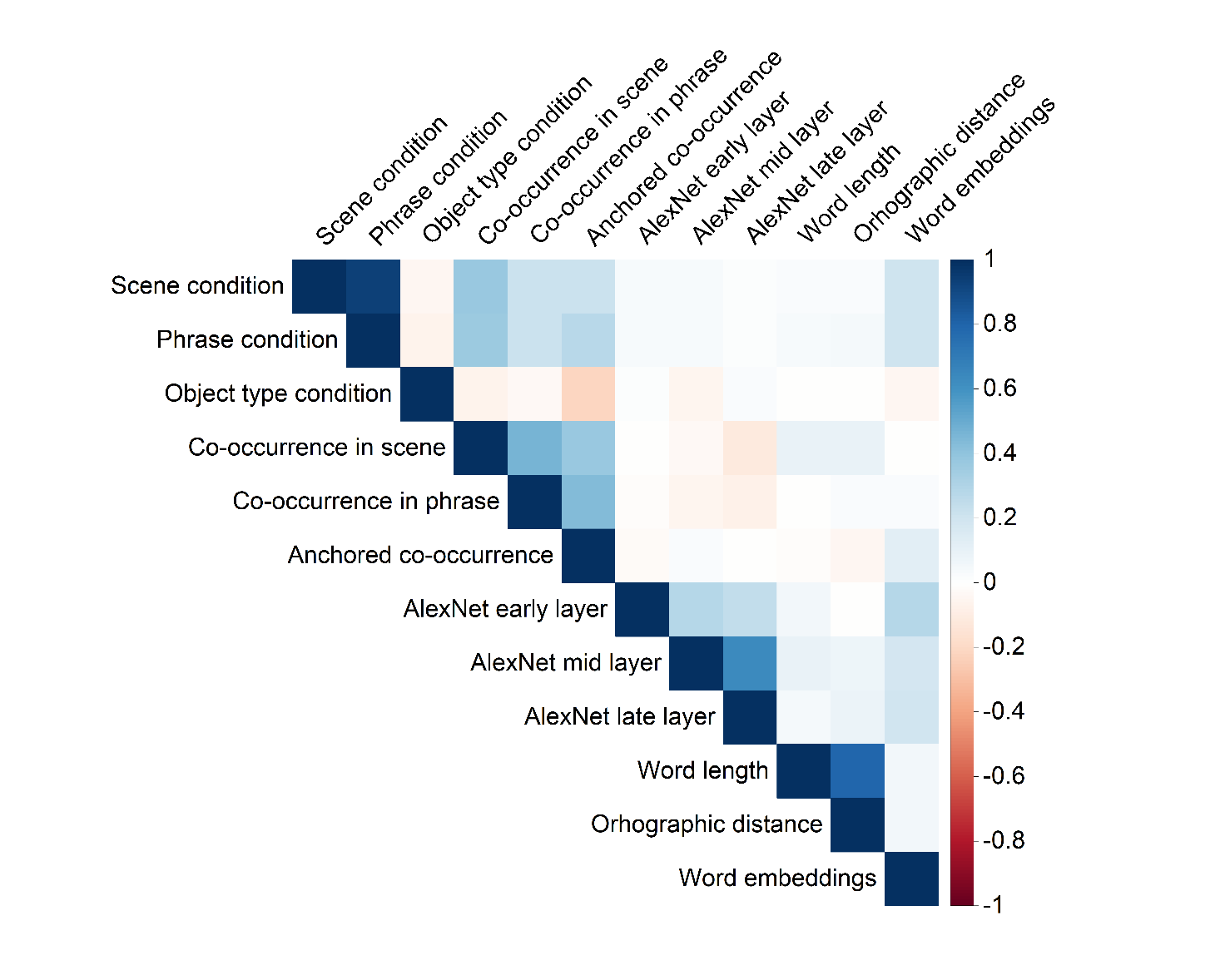
**

**Sup. Table 1 –** Variance Inflation Factors (VIFs) for the predictors used in the main model

| **Predictors** | **VIF** |
| --- | --- |
| Modality (Words – Objects) | 3.645 |
| Object type condition | 1.065 |
| Phrase condition | 1.291 |
| Anchored co-occurrence | 1.464 |
| Co-occurrence in scene | 1.525 |
| Co-occurrence in phrase | 1.425 |
| AlexNet early layer | 1.184 |
| AlexNet mid layer | 1.768 |
| AlexNet late layer | 1.771 |
| Word length | 2.821 |
| Orthographic distance | 2.841 |
| Word embeddings | 1.189 |
| Modality x Object type cond | 1.084 |
| Modality x Phrase cond | 3.909 |
| Modality x Anchored co-oc | 1.450 |
| Modality x Co-oc in scene | 1.506 |
| Modality x Co-oc in phrase | 1.386 |
| Modality x AlexNet early layer | 1.190 |
| Modality x AlexNet mid layer | 1.771 |
| Modality x AlexNet late layer | 1.789 |
| Modality x Word length | 2.929 |
| Modality x Orth distance | 2.943 |
| Modality x Word embeddings | 1.178 |

**
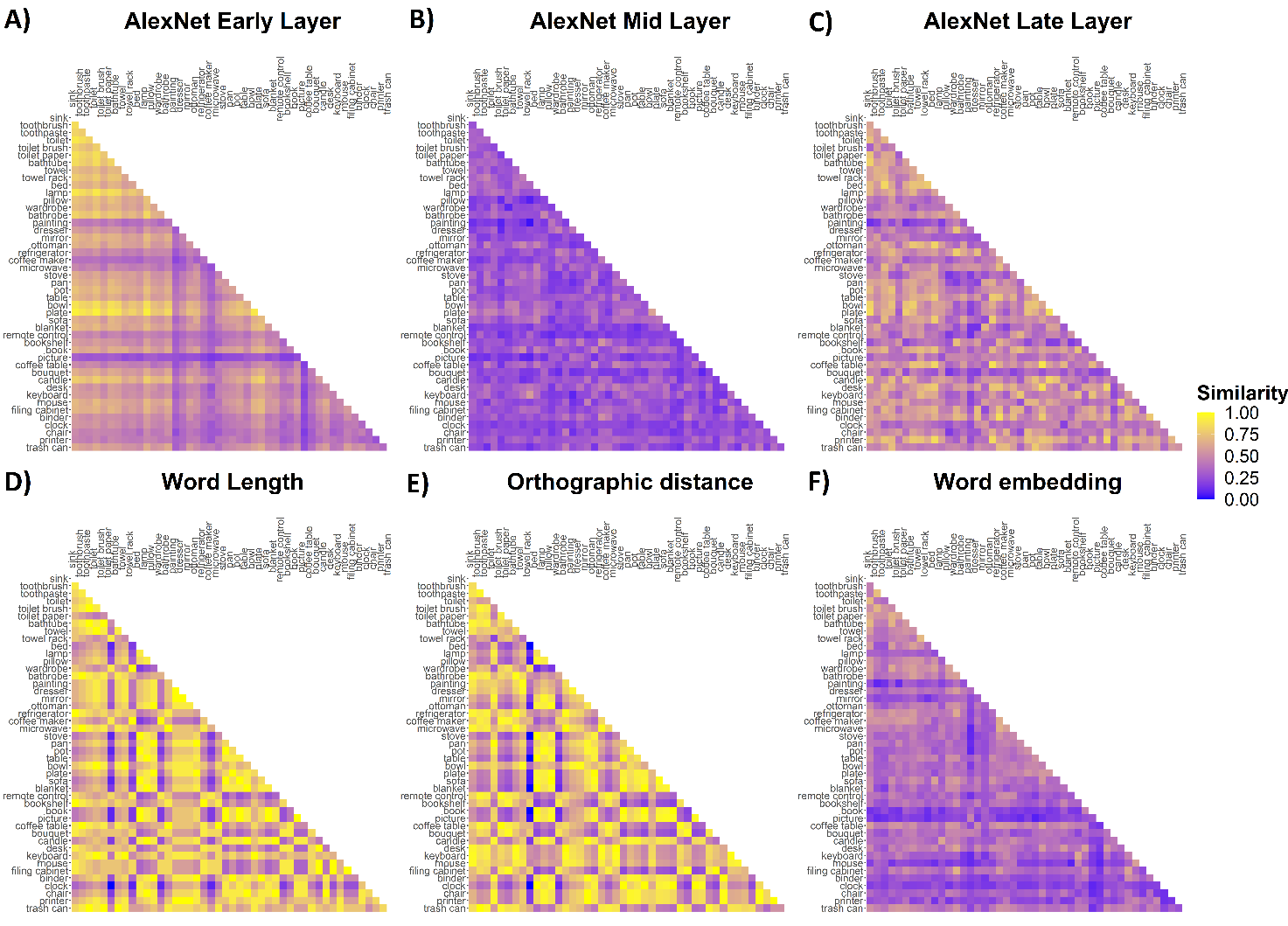
Sup. Fig. 2 –** Representational (Dis)similarity Matrices (RDMs) for the visual covariates for pictures (A, B and C) and for the orthographic and distributional semantics covariates for words (D, E and F). In A, B, C and D, colours represent the correlation between vectors (blue = 0 no correlation, yellow = 1 maximal correlation). In E and F, absolute value of the difference between word length / old20 of the pair is normalized to span between 0 (blue, bigger difference) to 1 (yellow, smaller difference).

**Supplementary Materials 2 – Results of the main model**

**Sup. Table 2 –** Results of the GLMM

| **Predictors** | **β** | **SE** | **z** | **p** |
| --- | --- | --- | --- | --- |
| (Intercept) | -0.321 | 0.065 | -4.809 | **<0.001** |
| Modality (Words – Objects) | -0.107 | 0.031 | -3.448 | **0.001** |
| Object type condition (Same – Different) | 0.245 | 0.048 | 5.106 | **<0.001** |
| Phrase condition (Same – Different) | 0.270 | 0.128 | 2.111 | **0.035** |
| Scene condition (Same – Different) | 1.078 | 0.075 | 14.474 | **<0.001** |
| Anchored co-occurrence | 0.005 | 0.028 | 0.165 | 0.869 |
| Co-occurrence in scene | 0.397 | 0.029 | 13.922 | **<0.001** |
| Co-occurrence in phrase | 0.063 | 0.028 | 2.292 | **0.022** |
| AlexNet early layer | -0.133 | 0.025 | -5.317 | **<0.001** |
| AlexNet mid layer | 0.026 | 0.031 | 0.846 | 0.397 |
| AlexNet late layer | 0.126 | 0.031 | 4.078 | **<0.001** |
| Word length | 0.049 | 0.039 | 1.271 | 0.204 |
| Orthographic distance | -0.050 | 0.039 | -1.270 | 0.204 |
| Word embeddings | 0.338 | 0.025 | 13.363 | **<0.001** |
| Modality x Object type condition | -0.006 | 0.034 | -0.181 | 0.857 |
| Modality x Phrase condition | 0.117 | 0.087 | 1.346 | 0.178 |
| Modality x Scene condition | -0.280 | 0.050 | -5.601 | **<0.001** |
| Modality x Anchored co-occurrence | 0.009 | 0.019 | 0.498 | 0.619 |
| Modality x Co-occurrence in scene | -0.124 | 0.019 | -6.361 | **<0.001** |
| Modality x Co-occurrence in phrase | 0.018 | 0.019 | 0.967 | 0.334 |
| Modality x AlexNet early layer | 0.022 | 0.017 | 1.242 | 0.214 |
| Modality x AlexNet mid layer | 0.007 | 0.022 | 0.333 | 0.739 |
| Modality x AlexNet late layer | -0.112 | 0.022 | -5.157 | **<0.001** |
| Modality x Word length | 0.082 | 0.028 | 2.977 | **0.003** |
| Modality x Orthographic distance | 0.008 | 0.028 | 0.302 | 0.763 |
| Modality x Word embeddings | -0.034 | 0.018 | -1.932 | 0.053 |

**Sup. Fig. 3 –** Model-estimated effects of the covariates on pairwise similarity ratings for object pictures and words. Colours of points reflect the values of pairs for the given predictor and match the ones in the RDMs showed above. Stimulus modality is indicated by x-axis position (left = objects, right = words). Points reflect estimated similarity for each pair of objects averaged across all the different contexts (i.e., the third object a triplet) in which they were presented. 95 % confidence interval are represented by the shaded area around lines for continuous predictors.

**
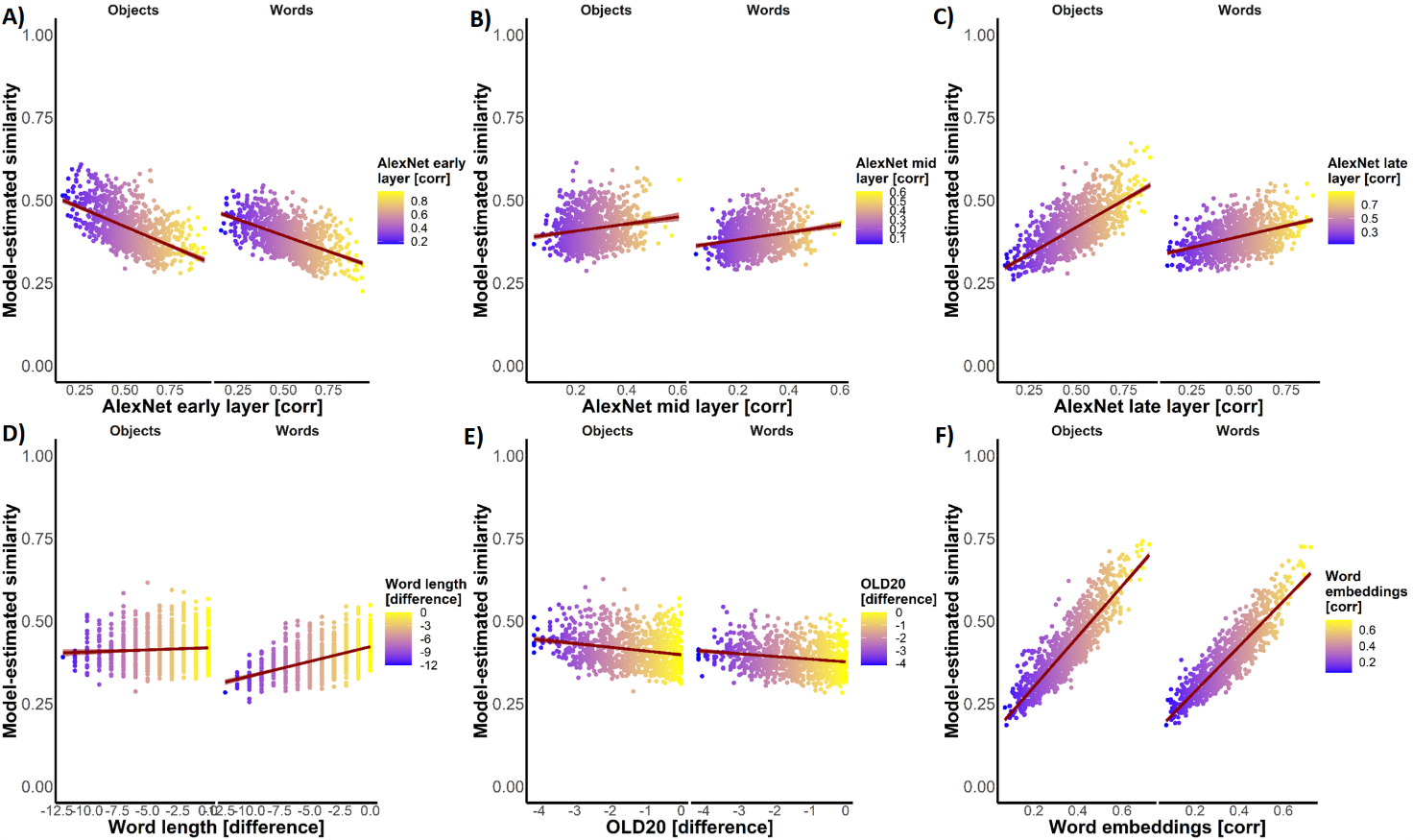
**

**Supplementary Materials 3 – Factor correlations and VIFs in the model with ratings**

We explored what makes anchor objects different from local objects (as seen from the effect of the *Object type condition* predictor), comparing this division with the ratings we collected in a separate experiment. First of all, we organized our ratings of *moveability*, *manipulability* and *real-world size* in an RDM format (similarity values were computed as the absolute value of the difference between the two values of each pair, as done for e.g., word length). We then computed pairwise correlations between each of the ratings RDMs and the object type condition RDM. We found that object type condition had a strong correlation with real-world size (r = 0.713) and moveability (r = 0.639), with the two measures also being strongly correlated (r = 0.659). On the other hand, manipulability did not show to have strong correlation with either object type condition (r = -0.042), or moveability (r = -0.065) and real-world size (r = -0.082).

**Sup. Fig. 4 –** Matrix of correlations between the ratings and the object type condition factor

**
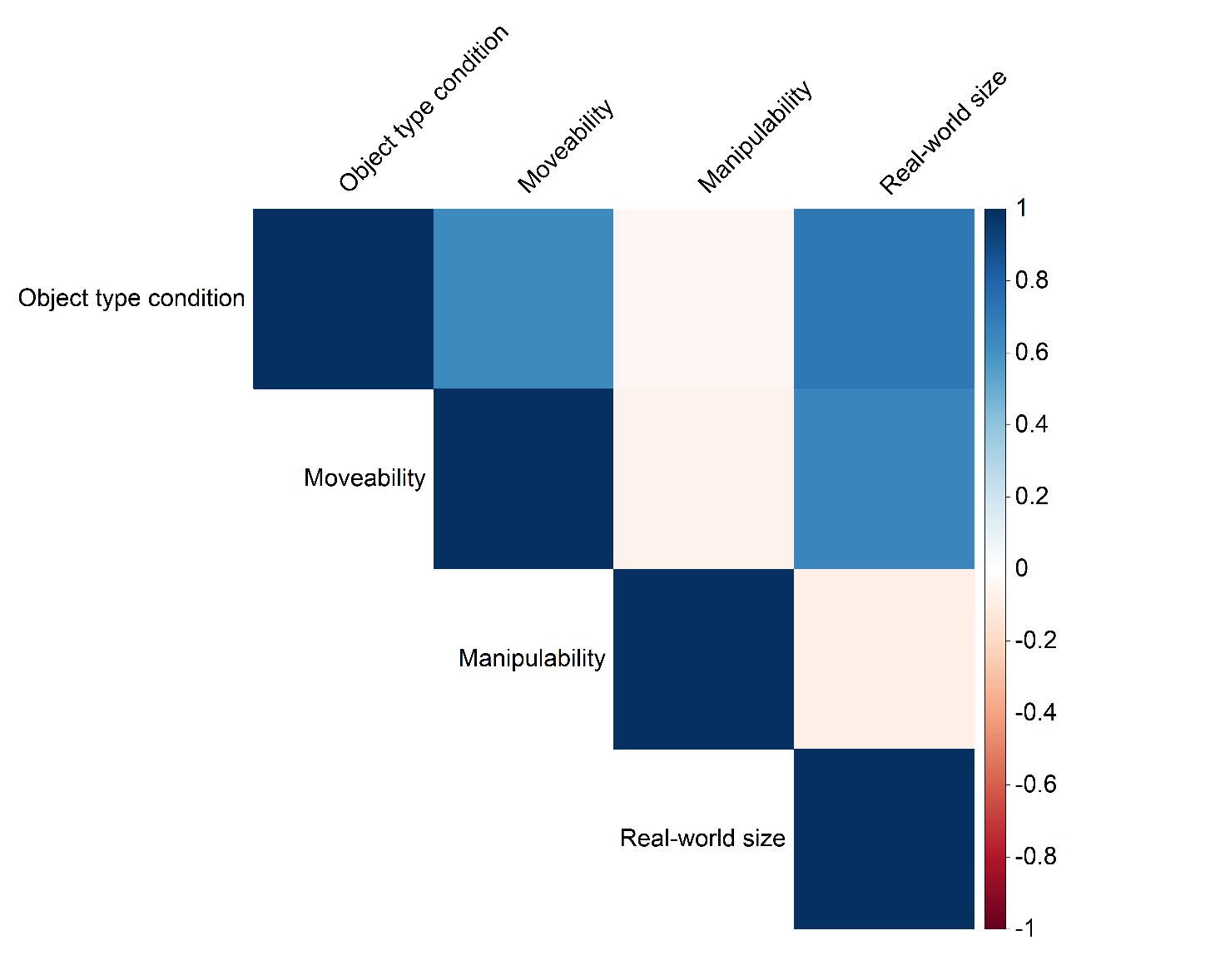
**

Second, we implemented another GLMM modeling the data with the same structure of fixed and random factors, but adding also the three rating predictors:

$$behavioral similarity \sim stimulus modality*(scene similarity+phrase similarity+object type similarity+cooccurrence in scene+cooccurrence in phrase +anchored cooccurrence +\boldsymbol{ratings}+ covariates)$$

$$+\left( 1 \right| pairs)+\left( 1 \right| context objects)$$

This new model including the ratings had a significantly better fit compared to the previous one without those measures (AIC difference = 57, *χ^2^* = 58.528, *p* < 0.001), and despite the new model being more complex in terms of number of parameters. The model also did not show problematic levels of multicollinearity, when inspecting the VIFs of each term.

**Sup. Table 1 –** Variance Inflation Factors (VIFs) for the predictors used in the model including rating measures

| **Predictors** | **VIF** |
| --- | --- |
| Modality (Words – Objects) | 3.651 |
| Moveability | 2.064 |
| Real-world size | 2.518 |
| Manipulability | 1.027 |
| Object type condition | 2.359 |
| Phrase condition | 1.300 |
| Anchored co-occurrence | 1.535 |
| Co-occurrence in scene | 1.559 |
| Co-occurrence in phrase | 1.431 |
| AlexNet early layer | 1.234 |
| AlexNet mid layer | 1.769 |
| AlexNet late layer | 1.776 |
| Word length | 2.866 |
| Orthographic distance | 2.886 |
| Word embeddings | 1.197 |
| Modality x Moveability | 2.049 |
| Modality x Real-world size | 2.505 |
| Modality x Manipulability | 1.029 |
| Modality x Object type condition | 2.308 |
| Modality x Phrase condition | 3.940 |
| Modality x Anchored co-occur. | 1.535 |
| Modality x Co-occur. in scene | 1.538 |
| Modality x Co-occur. in phrase | 1.392 |
| Modality x AlexNet early layer | 1.247 |
| Modality x AlexNet mid layer | 1.772 |
| Modality x AlexNet late layer | 1.794 |
| Modality x Word lenght | 2.974 |
| Modality x Orthographic distance | 2.993 |
| Modality x Word embeddings | 1.190 |

**Sup. Fig. 5 –** Representational (Dis)similarity Matrices (RDMs) for the a priori object type distinction (A), and for the object features ratings (B, C and D). Every cell represents pairwise similarity for that given dimension. In A yellow represents pairs of objects that belong to the same type (maximal similarity), while blue represents pairs that belong to different types (minimal similarity). In B, C and D, absolute value of the difference between ratings of the pair is normalized to span between 0 (blue, bigger difference) to 1 (yellow, smaller difference).


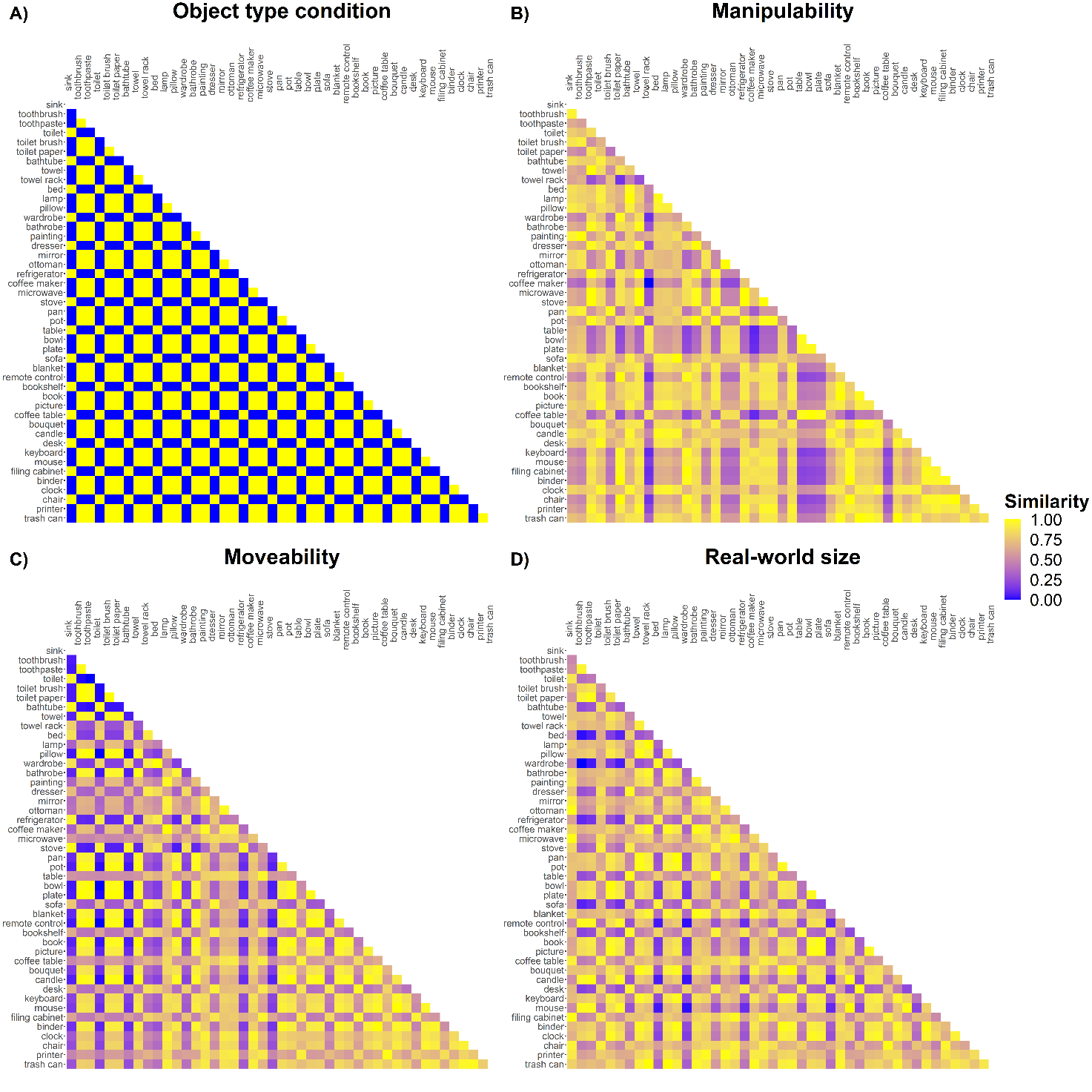


**Supplementary Materials 4 – Model with ratings measures**

Results overall resembled the one from the previous model, but with some important differences. First, adding the rating measures, the main effect of Object type condition got strongly reduced and was no longer significant (β=0.120, SE=0.071, z=1.681, p=0.093). On the other hand, we found significant main effects of the newly introduced moveability (β=0.079, SE=0.033, z=2.386, p=0.017) and manipulability measure (β=0.046, SE=0.023, z=1.984, p=0.047), both showing that pairs that are similar along those dimensions are also more likely to be judge more similar behaviourally. Real-world size did not show a significant main effect (β=0.022, SE=0.037, z=0.587, p=0.557), but resulted in having a significant interaction with stimulus modality (β=-0.057, SE=0.026, z=-2.158, p=0.031), with a stronger effect of this dimension on behavioural similarity for object pictures than for words. Similarly, manipulability had a significant interaction with stimulus modality (β=-0.111, SE=0.017, z=-6.713, p<0.001), having a stronger effect on perceived similarity for object pictures than for words.

**Sup. Table 4 –** Results of the GLMM including object features ratings

| **Predictors** | **β** | **SE** | **z** | **p** |
| --- | --- | --- | --- | --- |
| (Intercept) | -0.314 | 0.065 | -4.853 | **<0.001** |
| Modality (Words – Objects) | -0.101 | 0.031 | -3.252 | **0.001** |
| Moveability | 0.079 | 0.033 | 2.386 | **0.017** |
| Real-world size | 0.022 | 0.037 | 0.587 | 0.557 |
| Manipulability | 0.046 | 0.023 | 1.984 | **0.047** |
| Object type condition (Same – Different) | 0.120 | 0.071 | 1.681 | 0.093 |
| Phrase condition (Same – Different) | 0.249 | 0.128 | 1.951 | 0.051 |
| Scene condition (Same – Different) | 1.065 | 0.074 | 14.339 | **<0.001** |
| Anchored co-occurrence | 0.015 | 0.028 | 0.544 | 0.586 |
| Co-occurrence in scene | 0.387 | 0.029 | 13.488 | **<0.001** |
| Co-occurrence in phrase | 0.060 | 0.028 | 2.184 | **0.029** |
| AlexNet early layer | -0.119 | 0.025 | -4.658 | **<0.001** |
| AlexNet mid layer | 0.024 | 0.031 | 0.793 | 0.428 |
| AlexNet late layer | 0.122 | 0.031 | 3.971 | **<0.001** |
| Word length | 0.043 | 0.039 | 1.101 | 0.271 |
| Orthographic distance | -0.048 | 0.039 | -1.227 | 0.220 |
| Word embeddings | 0.343 | 0.025 | 13.554 | **<0.001** |
| Modality x Moveability | 0.019 | 0.024 | 0.807 | 0.420 |
| Modality x Real-world size | -0.057 | 0.026 | -2.158 | **0.031** |
| Modality x Manipulability | -0.111 | 0.017 | -6.713 | **<0.001** |
| Modality x Object type condition | 0.039 | 0.049 | 0.791 | 0.429 |
| Modality x Phrase condition | 0.153 | 0.087 | 1.750 | 0.080 |
| Modality x Scene condition | -0.267 | 0.050 | -5.322 | **<0.001** |
| Modality x Anchored co-occurrence | 0.001 | 0.020 | 0.057 | 0.955 |
| Modality x Co-occurrence in scene | -0.116 | 0.020 | -5.870 | **<0.001** |
| Modality x Co-occurrence in phrase | 0.021 | 0.019 | 1.107 | 0.268 |
| Modality x AlexNet early layer | 0.021 | 0.018 | 1.184 | 0.236 |
| Modality x AlexNet mid layer | 0.010 | 0.022 | 0.456 | 0.648 |
| Modality x AlexNet late layer | -0.114 | 0.022 | -5.267 | **<0.001** |
| Modality x Word length | 0.085 | 0.028 | 3.037 | **0.002** |
| Modality x Orthographic distance | 0.016 | 0.028 | 0.555 | 0.579 |
| Modality x Word embeddings | -0.036 | 0.018 | -2.025 | **0.043** |

**
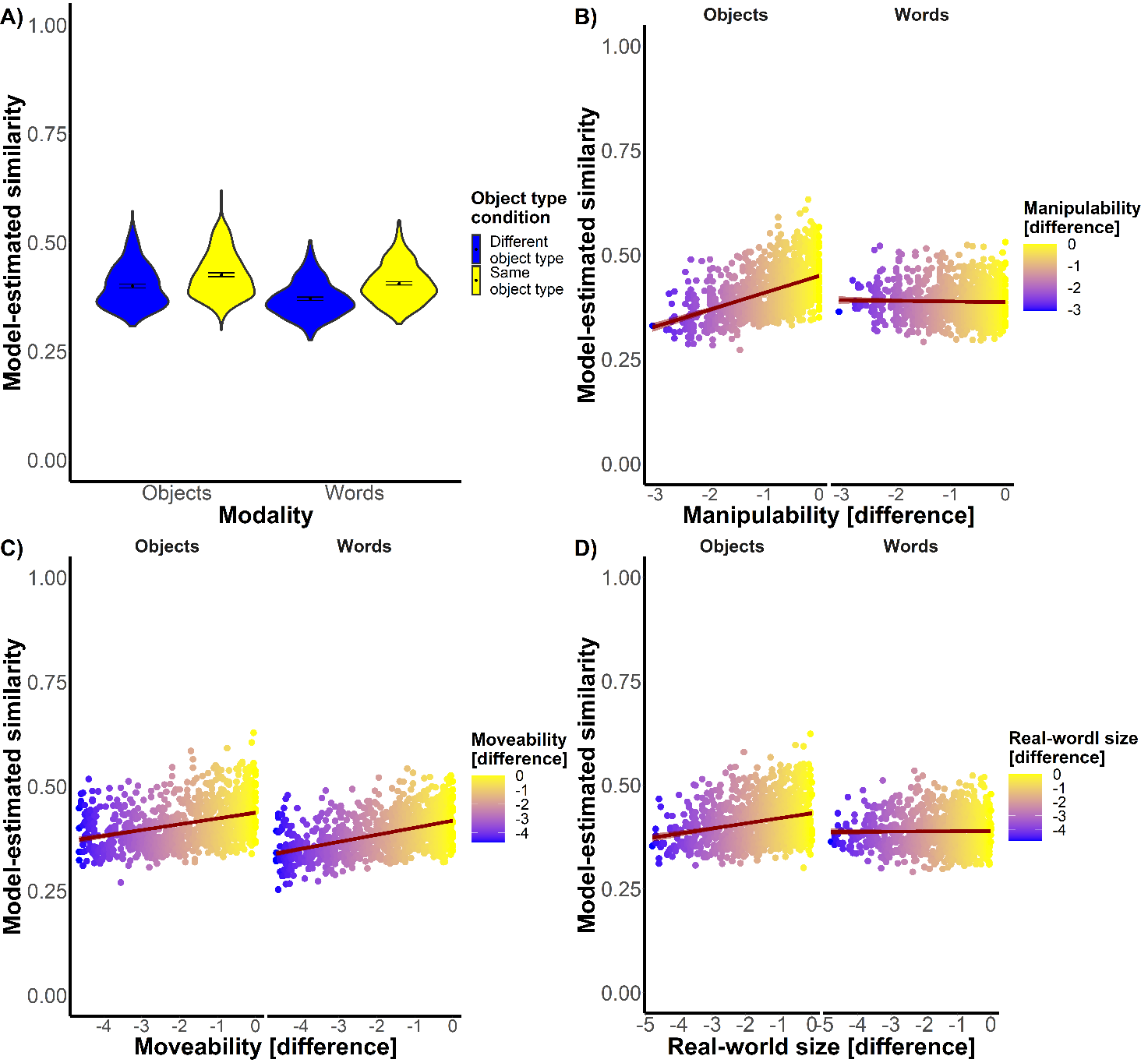
Sup. Fig. 6 –** Model-estimated effects of the object type condition predictor as well as for the object features ratings, estimated from the model including the ratings themselves. Colours of violins and points reflect the values of pairs for the given predictor and match the ones in the RDMs showed above. Stimulus modality is indicated by either x-axis position (left = objects, right = words). Points and violins reflect estimated similarity for each pair of objects averaged across all the different contexts (i.e., the third object a triplet) in which they were presented. 95 % confidence interval are represented by error bars in the violins (point is the mean), and by the shaded area around lines for continuous predictors.
